# Advanced Research of Abdominal Aortic Aneurysms on Metabolism

**DOI:** 10.3389/fcvm.2021.630269

**Published:** 2021-02-05

**Authors:** Yangfeng Hou, Wenjun Guo, Tianfei Fan, Bolun Li, Weipeng Ge, Ran Gao, Jing Wang

**Affiliations:** State Key Laboratory of Medical Molecular Biology, Institute of Basic Medicine, Chinese Academy of Medical Sciences, School of Basic Medicine, Peking Union Medical College, Beijing, China

**Keywords:** abdominal aortic aneurysm, metabolism, diabetes mellitus, hyperlipidemias, pathogenesis

## Abstract

Abdominal aortic aneurysm (AAA) is a cardiovascular disease with a high risk of death, seriously threatening the life and health of people. The specific pathogenesis of AAA is still not fully understood. In recent years, researchers have found that amino acid, lipid, and carbohydrate metabolism disorders play important roles in the occurrence and development of AAA. This review is aimed to summarize the latest research progress of the relationship between AAA progression and body metabolism. The body metabolism is closely related to the occurrence and development of AAA. It is necessary to further investigate the pathogenesis of AAA from the perspective of metabolism to provide theoretical basis for AAA diagnosis and drug development.

## Introduction

Abdominal aortic aneurysm (AAA) is a disease in which the abdominal aorta gradually expands like a tumor under the action of blood pressure. Usually, when the diameter of the abdominal aorta exceeds 3 cm or increases by more than 50% compared with normal, it can be diagnosed with AAA (1). AAA commonly occurs in elderly male over age of 65, and the morbidity can reach 8% (1). The most serious consequence is the rupture of the artery wall due to it cannot withstand the impact of blood flow. The mortality from ruptured aneurysms can exceed 80% (1, 2), and the mortality of patients undergoing repair surgery still exceeds 50% (1, 3, 4).

AAA is caused by the destruction of the abdominal aorta, especially the elastin break, due to a variety of congenital or acquired factors. Previous studies have found that the pathological process of AAA mainly includes local inflammatory cell infiltration, protease hydrolysis of elastic fibers and collagen fibers, vascular smooth muscle cell (VSMC) apoptosis and phenotypic transformation, and oxidative stress caused by oxidation and anti-oxidation imbalance (1, 2, 5). In recent years, researchers have discovered that the metabolic disorders of amino acids, lipids and glucose *in vivo* are closely related to the occurrence and development of AAA by affecting the above-mentioned pathological processes. This article will review the latest research of the relationship between AAA and the metabolism of main nutrients *in vivo*.

## Amino Acid Metabolism and AAA

### Homocysteine

Homocysteine (Hcy), an intermediate product in the metabolism of methionine and cysteine, is a sulfur-containing amino acid. Lack of key enzymes required for Hcy metabolism, such as cystathionine β-synthase (CBS), and coenzymes, such as folic acid, vitamin B6, and vitamin B12, will cause hyperhomocysteinemia (HHcy).

HHcy is a risk factor for many cardiovascular diseases, including AAA (6–8). Early clinical studies found that HHcy caused by congenital CBS deficiency would increase the risk of vascular diseases, such as abdominal aortic aneurysm, pulmonary embolism, and myocardial infarction, and lowering of circulating Hcy level by long-term treatment can significantly improve the vascular outcome of patients with CBS deficiency (9, 10). One of the most representative drugs treating HHcy and entering phase 1/2 clinical trials is OT-58, which is obtained from human CBS (11). Some preclinical studies provide evidence that OT-58 may be further investigated in AAA treatment in the future (12, 13).

**Figure 1:**
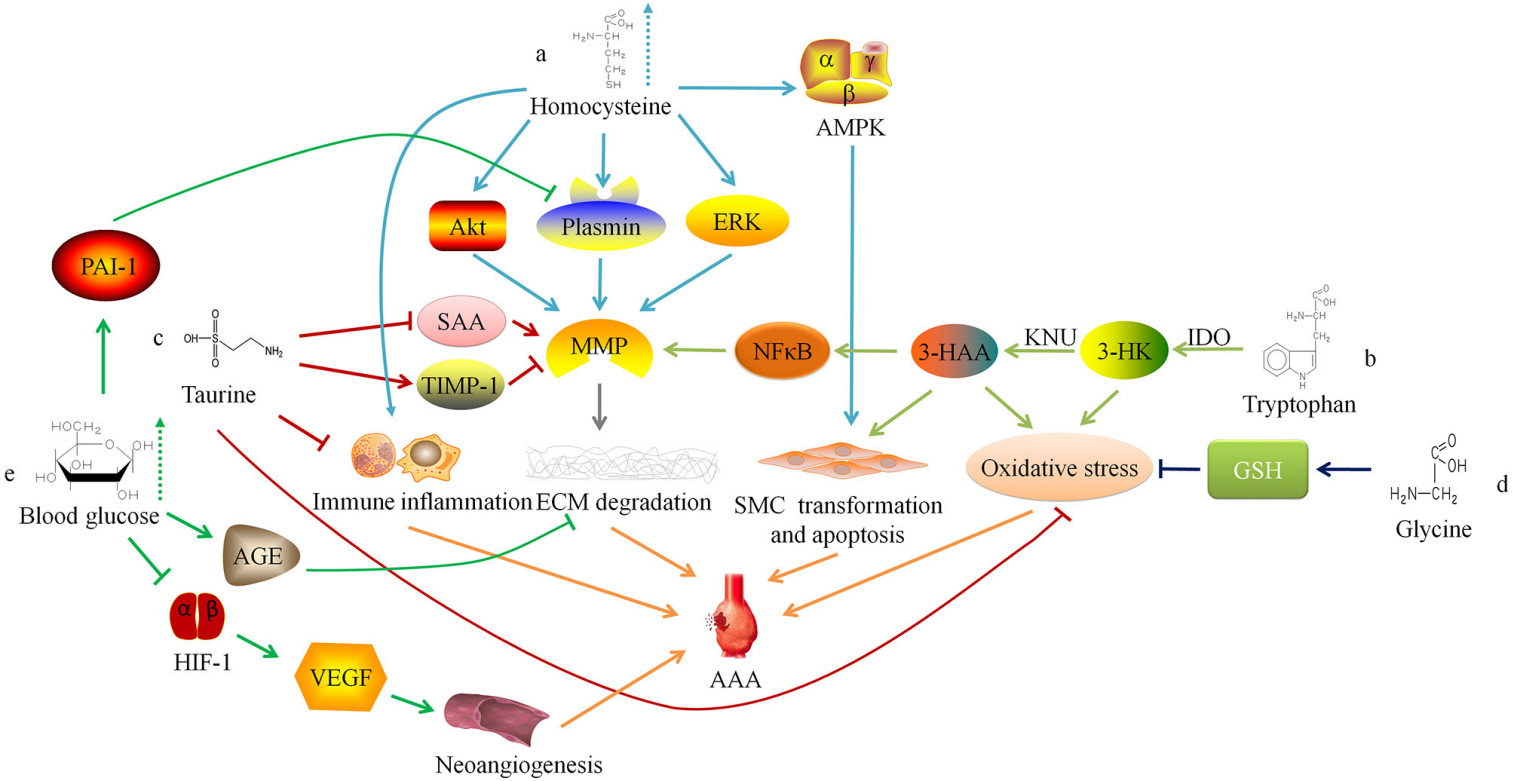
Relationship between amino acid and glucose metabolism and abdominal aortic aneurysm. ERK, extracellular regulated protein kinases; MMP, matrix metalloproteinase; ECM, extracellular matrix; AAA, abdominal aortic aneurysm; AMPK. AMP-activated protein kinase; SMC, smooth muscle cell; IDO, indoleamine 2,3-dioxygenase; 3-HK, 3-hydroxykynurenine; KNU, kynureninase; 3-HAA, 3-hydroxyanthranilic acid; NFκB, nuclear factor kappa B; SAA, serum amyloid A; TIMP-1, tissue inhibitor of matrix metalloproteinase-1; GSH, Glutathione; AGE, advanced glycation end-product; HIF-1, hypoxia-inducible factor-1; VEGF, vascular endothelial growth factor.

HHcy may also increase the concentration and activity of MMP-9 in the blood vessel wall by activating ERK and Akt signaling pathways to promote the occurrence and development of AAA (14, 15). Siennicka et al. (16) found that high concentrations of Hcy can participate in the pathological process of AAA by affecting proteolysis and coagulation/fibrinolysis system. In AAA with thin intraluminal thrombus, high Hcy can increase matrix metalloproteinase (MMP)-2 and fibrinolytic factors (plasminogen and tissue-type plasminogen activator), leading to the degradation of elastin and collagen. On the contrary, in AAA with thicker thrombus, high level of Hcy produce the opposite effect, which may be one of reasons that lesion with thicker thrombus is less likely to rupture, and intraluminal thrombus may have some influence on the effect of HHcy on AAA (16). Besides, HHcy can also cause local inflammatory cell infiltration in AAA, increase the expression of inflammatory factors (such as IL-6), and promote the transformation of VSMC phenotype from contractile to synthetic (17). Moreover, HHcy can induce autophagy in VSMC by activating the AMPK signaling pathway, which is characterized by the increased expression of autophagy-related proteins LC3 and Beclin-1 (Figure 1a) (17).

Researchers have confirmed that serum Hcy level is positively correlated with the size, diameter, expansion rate, and risk of rupture of aneurysms in AAA patients (18–22). Therefore, some researchers have tried to inhibit the occurrence and development of AAA by regulating the metabolism of Hcy. Fan et al. (23) found that excessive supplementation of Hcy's metabolic precursor methionine can induce Hcy production and cause hypermethioninemia (HMET), thereby enhancing the expression of MMP-2 and inflammatory response in the vessel wall and exacerbating the development of AAA in rats. This suggests that for AAA patients, restricting the intake of methionine may have a protective effect. There is also preliminary evidence in clinical practice that the low-methionine diet combined with vitamin B6, vitamin B12 and folic acid can reduce the concentration of serum Hcy for HHcy patients with AAA, and may protect people from AAA (10, 24).

### Tryptophan

Tryptophan (Trp), one of the essential amino acids, is catalyzed by indoleamine 2,3-dioxygenase (IDO) or tryptophan 2,3-dioxygenase (TDO) to generate kynurenine (Kyn). The catabolism of Kyn can produce 3-hydroxykynurenine (3-HK) and 3-hydroxyanthranilic acid (3-HAA). 3-HAA can be produced from 3-HK under the action of kynureninase (KNU).

Wang et al. (25) found that the degradation of the elastic laminae and arterial expansion rate in Apoe^−/−^ IDO^−/−^ mice was significantly reduced. Further exploration of the mechanism found that 3-HAA up-regulated the expression of MMP-2 by activating the transcription factor nuclear factor kappa B (NFκB). Therefore, knockdown of KNU in mice can inhibit the production of 3-HAA and MMP-2, thereby inhibiting the formation of AAA. The detection of human AAA tissue samples also found that the anti-3-HAA, anti-IDO and anti-KNU antibody staining intensity in aneurysm tissue was stronger than that in non-aneurysm tissue sections (25). These results suggest that reducing the serum 3-HAA level in AAA patients by regulating Trp metabolism may be a potential therapeutic target. Furthermore, 3-HK and 3-HAA can generate free radicals (26), which may lead to an imbalance between oxidation and anti-oxidation in the blood vessel wall and promote AAA. Metghalchi et al. (27) found that IDO deficiency may limit the development of aneurysms by reducing the production of 3-HAA, but the specific mechanism needs further research (Figure 1b). At present, several IDO inhibitors, such as Indoximod, Epacadostat, and Navoximod, have been approved for patients in anti-tumor therapy, but there is a lack of research to assess the application of these drugs on AAA (28).

### Taurine

Taurine (Tau) is an amino acid converted from sulfur-containing amino acids. Kim et al. (29) found that oral supplementation of Tau could inhibit AAA formation in mice, because Tau can react with the oxidant (such as HOCl) catalyzed by myeloperoxidase to alleviate oxidative stress, reduce inflammatory cell aggregation, and inhibit activity of matrix metalloproteinase-1 in the vessel wall. In addition, Tau can also reduce the serum amyloid A level (29), which can promote the AngII-induced formation of AAA in mice (30). These findings suggest that supplementing patients with Tau may be a potential prevention or treatment method for AAA (Figure 1c).

Besides, Tau can also bind with ursodeoxycholic acid to form tauroursodeoxycholic acid (TUDCA). By animal experiments, researchers found that TUDCA can reduce the apoptosis of VSMC by inhibiting endoplasmic reticulum stress, thereby reducing the maximum diameter of aneurysms induced by AngII (31).

### Glycine

Glycine (Gly) can form the endogenous antioxidant glutathione (GSH) with glutamic acid and cysteine. Studies have found that Gly has protective effects on cardiovascular diseases, such as antagonizing cardiac and cerebral ischemic damage (32, 33), and lowering blood pressure (34, 35). The researchers found that supplementing the diet with an appropriate dose of Gly could prevent cardiovascular diseases (36, 37), which may depend on potential mechanisms showing below: (1) Activating the glycine-gated Cl^−^ channel, hyperpolarizing the cell membrane, preventing the influx of Ca^2+^, and inhibiting the effect of Ca^2+^ on the growth and migration of endothelial cells (38); (2) Reducing the oxidation of nitric oxide through a glutathione-dependent mechanism, thereby increasing its content in the circulatory system (39); (3) Increasing the synthesis of glutathione in vascular inherent cells, thus playing a cytoprotective role (40).

Lack of Gly or mutations in the coding gene may result in decreased synthesis of elastin and collagen or abnormal structure, by which the blood vessel wall will become weak and gradually expand to form aneurysms under the pressure of blood flow (41). Moreover, studies have reported that glycine inhibits the production of reactive oxygen species (ROS) by synthesizing GSH to reduce the oxidative stress response of the blood vessel wall (42), and Gly can also regulate glucose and lipid metabolism (35, 43, 44). These findings suggest that glycine is involved in the pathophysiological progress of AAA, and further research is needed to study the mechanism (Figure 1d).

## Glycometabolism and AAA

Glycometabolism refers to a series of complex chemical reactions of carbohydrates such as glucose and glycogen *in vivo*. Diabetes mellitus (DM) is a group of metabolic diseases characterized by hyperglycemia. Although DM is an important risk factor for cardiovascular events and atherosclerosis-related diseases (45–47), epidemiological investigations have found that DM and fasting blood glucose is negatively related to AAA (48–50).

Miyama et al. (51) found that compared with normal blood glucose, AAA mice with hyperglycemia had less blood vessel expansion, arterial wall inflammatory cell infiltration, elastic fiber degradation and neoangiogenesis. Furthermore, the degree of AAA expansion and pathophysiological changes in diabetic mice treated with insulin were greater than those of diabetic AAA mice not treated with insulin, suggesting that hyperglycemia can limit the development of experimental aortic aneurysms. The mechanism may be linked to the down-regulation of vascular endothelial growth factor expression via interfering with the hypoxia-inducible factor-1 signaling pathway by hyperglycemia. Dua et al. (52) also found that hyperglycemia may also increase the level of endogenous plasminogen activator inhibitor-1, inhibit plasmin production, and reduce the infiltration of macrophages and the expression of MMPs in the arterial wall, thereby limiting the progress of experimental AAA.

Besides, chronic hyperglycemia can make extracellular matrix (ECM) glycation to form advanced glycation end-products (AGEs), which increases the stiffness of blood vessels (53). Koole et al. (54) found that the concentration of pentosidine, one of the AGEs, was negatively correlated with the diameter of the abdominal aorta in AAA patients with DM. The glycosylated AAA tissues can resist MMPs-induced degradation of type I collagen. Golledge et al. (55) incubated activated monocytes with glycosylated collagen fibers and found that glycosylated collagen tissue can inhibit monocytes from secreting MMPs. These findings indicate that AGEs induced by hyperglycemia may play a protective role in the progress of AAA (Figure 1e).

Clinical studies have also found that DM patients have a lower incidence of AAA (56), and AAA patients with DM have smaller aneurysms, lower expansion rate and are less likely to rupture (57, 58). In addition, some researchers used positron emission tomography/computed tomography to detect AAA tissue and found an increase in the uptake of 18F-fluorodeoxyglucose mediated by glucose transporters, suggesting enhanced glucose metabolism activity in the lesion tissue (59). Tsuruda et al. (60) found in mouse models that the enhancement of glycolytic activity in arterial wall tissue is one of the reasons for the development of AAA. They tried intraperitoneal administration of glycolysis inhibitor 2-deoxyglucose, a glucose analog and found that it can alleviate AAA via inhibiting macrophage survival and adhesion to endothelial cells, ECM degradation and inflammation. Later studies confirmed that the energy metabolism in macrophages changed from oxidative phosphorylation to glycolysis in the HHcy state, which promoted the production of pro-inflammatory cytokines and ROS, thereby aggravating AAA (61). These results suggest that interference with glycolytic activity may be a potential therapeutic target for AAA.

## Lipid Metabolism and AAA

Lipids, produced by the dehydration condensation of fatty acids and alcohols, refer to ester compounds and their derivatives, including triglycerides (TG) and lipoid (phospholipids, glycolipids, sterols, and their esters). Atherosclerosis (AS) and AAA have some similar pathophysiological processes, such as chronic inflammation, VSMC apoptosis and phenotypic transformation. Although it is still uncertain whether AS and AAA have a causal relationship (62), some traditional risk factors for AS (such as hyperlipemia, hypertension, smoking, age, gender, etc.) are also related to AAA (63–67). Investigations and studies have found that serum low density lipoprotein cholesterol (LDL-C), total cholesterol (TC), and TG levels are positively correlated with AAA (64–66). Nevertheless, whether elevated TG level will promote the occurrence and development of AAA is still controversial (68, 69).

**Figure 2:**
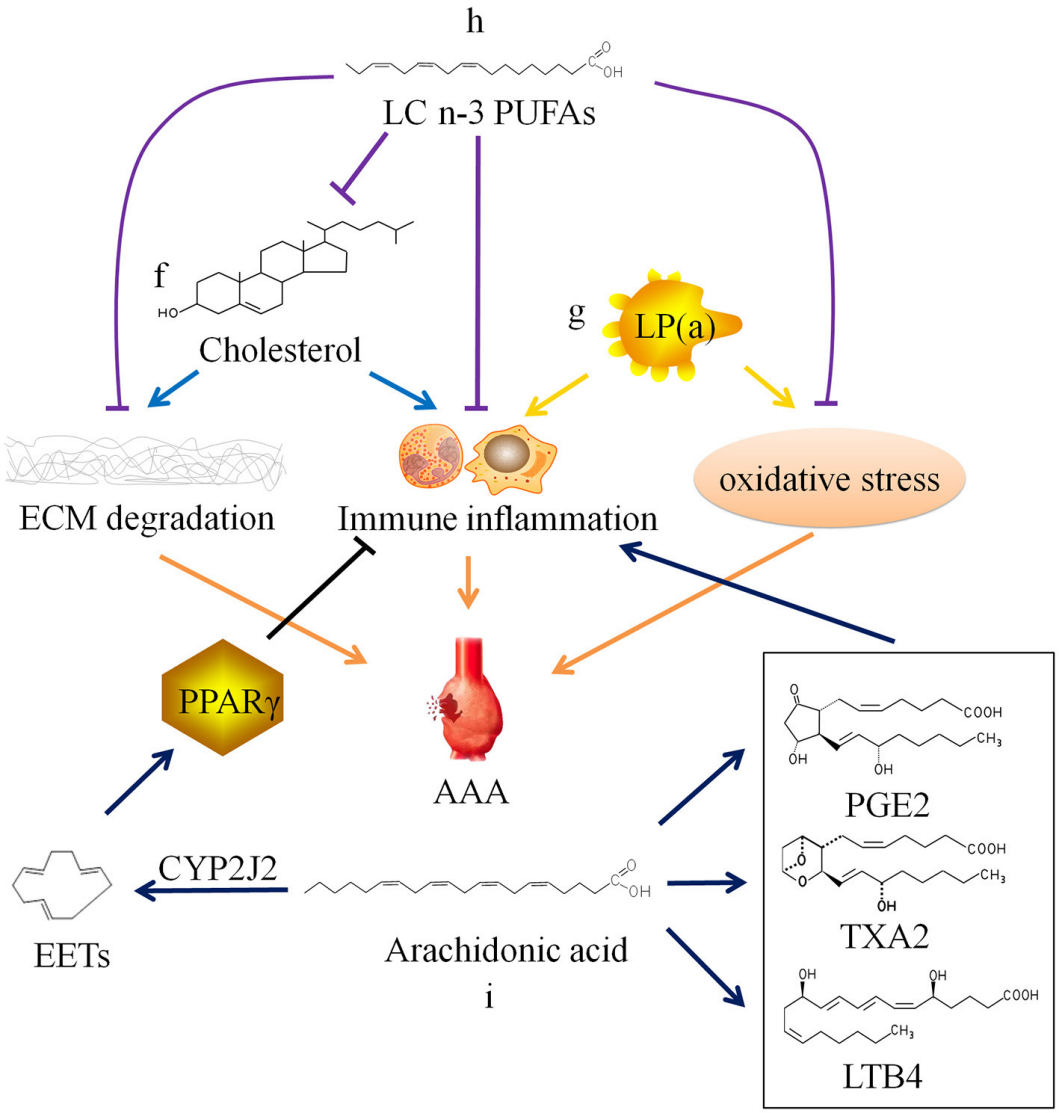
Relationship between lipid metabolism and abdominal aortic aneurysm. ECM, extracellular matrix; AAA, abdominal aortic aneurysm; SMC, smooth muscle cell; LP(a), lipoprotein(a); LC n-3 PUFAs, long chain omega-3 polyunsaturated fatty acids; PGE2, prostaglandin E2; TXA2, thromboxane A2; LTB4, leukotriene B4; CYP2J2, Cytochrome P450 epoxygenase 2J2; EETs, epoxyeicosatrienoic acids; PPARγ, peroxisome proliferator-activated receptor γ.

Cholesterol is often combined with lipoproteins in plasma, and low density lipoprotein (LDL) is the main carrier for transporting endogenous cholesterol. When TC and/or LDL-C abnormally elevated beyond the normal range, it will lead to hypercholesterolemia. Hobbs et al. (70) found through case-control analysis that there is a significant positive correlation between LDL-C and AAA, and hypothesized that LDL-C may cause AAA by inducing chronic inflammation-mediated degradation of ECM. Weng et al. (71) confirmed the causal relationship between high serum TC and LDL-C levels and AAA through Mendelian randomization study. Liu et al. (72) confirmed that hypercholesterolemia can promote the occurrence and development of AAA in mice. However, there is still a lack of basic research to clarify the specific cellular and molecular mechanisms of hypercholesterolemia on AAA (Figure 2f).

Contrary to LDL, high density lipoprotein (HDL) is mainly responsible for the reverse transport of cholesterol. HDL transports extrahepatic cholesterol to the liver, where cholesterol is transformed into bile acids and excreted, so that the high density lipoprotein cholesterol (HDL-C) level of hyperlipidemia is reduced. There is sufficient evidence to prove that HDL-C level is negatively correlated with AAA (73, 74), and the impaired cholesterol efflux caused by abnormal HDL transport function is also related to the development of AAA (75, 76). A recent study found that IgG anti-HDL antibody levels in AAA patients were elevated, and the antibody levels were positively correlated with the aortic diameter and negatively correlated with HDL-C levels, suggesting that AAA patients may have humoral immune response against HDL, which provides a new direction for the study of AAA pathogenesis and drug targets (77).

In addition, there is lipoprotein(a), also known as LP(a), in plasma which is related to AAA. LP(a) is a type of independent lipoprotein, which is produced by the liver and is not transformed into other lipoproteins, and its physiological function is currently unclear (78). Studies have shown that elevated LP(a) level is a risk factor for cardiovascular diseases. And current research is mainly about AS and thrombotic vascular diseases (79–82). Some researchers have also found that LP(a) levels are elevated in AAA patients (19). High plasma level of LP(a) may also be a risk factor for AAA (83), because LP(a) carries monocyte chemoattractant protein 1 and oxidized phospholipids, which can cause chronic inflammation and oxidative stress in the blood vessel wall (84–86), but the specific mechanism has not been confirmed (Figure 2g).

In recent years, it has been discovered that long chain polyunsaturated fatty acids (LCPUFAs) may be related to AAA. Arachidonic acid (ARA), a long chain omega-6 polyunsaturated fatty acid (LC n-6 PUFA), can be metabolized to produce prostaglandin E2, thromboxane A2, and leukotriene B4, which have been shown to aggravate AAA through their pro-inflammatory effect (87–90). The selective cyclooxygenase-2 (COX-2) inhibitor Celecoxib inhibits the formation of AAA in mice infused with AngII, which also confirms that the prostaglandin compounds produced by COX-2 catalyzed ARA play a certain role in AAA (91). Consistently, a clinical study conducted in Danish men showed that increased levels of ARA is related to AAA incidence and progression. AAA patients with high ARA levels were more likely to require surgical repair (92). Furthermore, this clinical study also found that the levels of eicosapentaenoic acid (EPA), a long chain omega-3 polyunsaturated fatty acid (LC n-3 PUFA), was not associated with AAA.

However, another Japanese clinical study found that EPA levels in AAA patients were relatively low compared with that in healthy person. There is a significant negative correlation between EPA levels or the EPA/arachidonic acid (ARA) ratio and AAA growth rate or maximum aneurysm diameter (93). A number of studies have found that LC n-3 PUFAs and their derivatives resolvins produced by enzymatic oxidation can inhibit the infiltration of inflammatory cells, the production of cytokines, the expression of MMPs, and oxidative stress (94–96). Moreover, LC n-3 PUFAs can reduce the synthesis of endogenous cholesterol and increase the metabolism of exogenous cholesterol, thereby reducing plasma TC level (97–99). *In vivo* experiments have confirmed that LC n-3 PUFAs and their derivatives can inhibit the occurrence and development of AAA (Figure 2h) (94, 100, 101). The contradiction between two clinical studies results from a huge difference on EPA diet of the populations. Furthermore, Cytochrome P450 cyclooxygenase 2J2 (CYP2J2) catalyzes the formation of epoxyeicosatrienoic acids (EETs) from ARA. Cai et al. (102) found that increased levels of EETs through CYP2J2 overexpression can activate peroxisome proliferator-activated receptor γ to exert anti-inflammatory effect, thereby preventing the development of AAA in mice (Figure 2i). These findings provide new ideas for the therapy of AAA.

Based on previous studies on the relationship between lipid metabolism and AAA, researchers are working on the development of preventive and therapeutic drugs for AAA from the perspective of regulating lipid metabolism. At present, the most widely used drug to regulate lipid metabolism is statins, the mechanism of which is to inhibit the rate-limiting enzyme 3-hydroxy-3-methylglutaryl coenzyme A reductase in endogenous cholesterol synthesis, thereby effectively reducing TC and LDL-C (103–105).

Moreover, proprotein convertase subtilisin/Kexin type 9 (PCSK9) inhibitors can reduce LDL-C by inhibiting the degradation of low density lipoprotein receptor (*Ldlr*), while PCSK9 gain-of-function mutation promotes AAA occurrence in mice (106). Previous clinical study has reported that PCSK9 inhibitors can reduce the risk of AAA (107). Evolocumab and Alirocumab, as PCSK9 inhibitors, have been approved for the clinical treatment of hyperlipidemia in many countries and regions (108, 109), but their actual effects on AAA have not been tested in clinical studies.

## Conclusions and Perspectives

Due to the concealment in the early stage and the lack of prevention and treatment methods, AAA is still a cardiovascular disease with high risk of death. Improving the diagnosis rate and cure rate, delaying the progression of AAA to prevent its rupture, and improving the prognosis are the goals that researchers and clinicians have been working on.

The above studies have found that the increase in plasma metabolites levels caused by abnormal amino acid and lipid metabolism is related to AAA. Therefore, clinicians can assess the risk of AAA formation by detecting the corresponding metabolites levels in plasma. For the elderly with a high incidence of AAA, clinicians can also provide dietary recommendations to achieve the purpose of early prevention. For patients who are diagnosed with AAA but have not yet met the surgical criteria or cannot tolerate surgery, drug therapy is still an important intervention (110). Although there is still a lack of recognized drugs that can treat AAA, experimental animal studies provide many new clues for potential drug targets.

In summary, under the general trend of multi-disciplinary cooperation, which leads to a number of new interdisciplinary sciences, it is in line with the trend of scientific development to study the pathogenesis and to develop therapeutic methods of AAA from a multi-disciplinary perspective. Moreover, with the rapid development of metabolomics in recent years, metabolomics methods have penetrated into many fields of medical science, including disease diagnosis and drug development. If the cellular and molecular mechanisms involved in the occurrence and development of AAA can be further elucidated from the perspective of metabolism, it will surely promote the application of metabolomics in the field of cardiovascular diseases. More specifically, it may provide new ideas and methods for early prevention, progression retardation and prognosis improvement of AAA.

## Author Contributions

YH wrote this review. WGu, TF, and RG modified and supplemented the content of the review. BL and WGe adjusted the grammar and structure of the article. JW advised on the article writing and reviewed the final version. All authors contributed to the article and approved the submitted version.

## Conflict of Interest

The authors declare that the research was conducted in the absence of any commercial or financial relationships that could be construed as a potential conflict of interest.
